# The Relationship Between Cybervictimization and Non-suicidal Self-Injury in Chinese Adolescents: A Moderated-Mediation Model

**DOI:** 10.3389/fpsyg.2020.572100

**Published:** 2021-02-15

**Authors:** Yulong Wang, Apian Chen, Hong Ni

**Affiliations:** ^1^Cognition and Human Behavior Key Laboratory of Hunan Province, School of Educational Science, Hunan Normal University, Changsha, China; ^2^Department of Psychology, California State University-Fresno, Fresno, CA, United States

**Keywords:** adolescent, non-suicidal self-injury, friendship quality, negative emotion, cybervictimization

## Abstract

The present study investigated the mediating role of negative emotion in the relationship between cybervictimization and non-suicidal self-injury (NSSI), and the moderating role of friendship quality in the indirect relationship. This model was tested with 1,326 Chinese adolescents who suffered from cyberbullying in the last 1 year; 727 were boys and 591 were girls, and their mean age was 13.67 years (*SD* = 1.34, range 11–17). Participants filled out questionnaires regarding cybervictimization, negative emotion, friendship quality, and non-suicidal self-injury. After demographic variables were controlled, cybervictimization was significantly positively associated with non-suicidal self-injury. Mediation analysis revealed that negative emotion partially mediated the association between cybervictimization and non-suicidal self-injury. Moderated mediation analysis further indicated that the mediated path was weaker for adolescents with higher levels of friendship quality. These findings underscore the importance of identifying the mechanisms that moderate the mediated path between cybervictimization and non-suicidal self-injury among adolescents.

## Introduction

Cyberbullying is a term used referring to the repeated dissemination of hostile or offensive information by electronic or digital media by individuals or groups in an attempt to cause psychological injury or discomfort to others. This behavior is characterized by invisibility and anonymity (Tokunaga, 2010). A transcultural study found that 8.8% were cybervictims, 3.1% were cyberaggressors, and 4.9% were cybervictims-cyberaggressors in Spain, whereas 8.7% were cybervictims, 5.1% were cyberaggressors, and 14.3% were cybervictims-cyberaggressors in Ecuador (Rodríguez-Hidalgo et al., 2020). The situation in China seems to be much more serious. According to a study of Chinese adolescents, 82.77% of adolescent respondents experienced cyberbullying at least once in the previous year, and 57.21% reported engaging in cyberbullying toward others more than once (Chen et al., 2016). Just like traditional bullying, cyberbullying is an intentional aggressive act that can be repeated and is designed to cause harm to the individual (Olweus, 2013). Of those who experience cyberbullying, 31% reported being extremely anxious, 10% felt very fearful, and 19% felt embarrassed (Raskauskas and Stoltz, 2007). In serious cases, cyberbullying has led to victims engaging in self-injurious behaviors (Hay and Meldrum, 2010).

Non-suicidal self-injury (NSSI) is a psychiatric behavior that is relatively common in adolescents and involves individuals intentionally and repeatedly physically harming themselves by engaging in behaviors such as cutting, burning, piercing, and hitting the wall. This behavior often is performed without the intent of committing suicide and is not accepted by society (Klonsky and Olino, 2008; Jiang et al., 2011). As a maladaptive coping strategy, NNSI has adverse consequences for adolescents’ mental health. The research showed that adolescents who frequently engage in self-injurious behaviors are more likely to experience depression and anxiety (Sho et al., 2009; Robinson et al., 2017) and have low self-esteem (Nock et al., 2009). The Diagnostic and Statistical Manual, Fifth Edition (DSM-5; American Psychiatric Association, 2013), categorized NSSI as an undiagnosed disorder; however, these behaviors have drawn increasing attention among those in the field of mental health. In the adolescent stage, NSSI has a development process with age (Barrocas et al., 2014), and there may be some gender difference (Gandhi et al., 2015). Both cross-sectional and longitudinal studies have confirmed that bullied adolescents are more likely to engage in NNSI than non-bullied adolescents, and as the rates of bullying increases so does the risk for NSSI (Jantzer et al., 2015; van Geel et al., 2015). As with traditional bullying, cybervictimization has been found to positively predict adolescents’ NNSI (Hay and Meldrum, 2010; Kessel Schneider et al., 2012; Wang et al., 2020).

Although prior research has identified a relationship between traditional bullying and adolescents’ NSSI, few studies have explored NSSI as a consequence of cyberbullying. Given the high incidence of adolescents’ cybervictimization and the deleterious consequences of NSSI, investigating their relationship could enhance the field’s understanding of the sequelae of cyberbullying and develop effective intervention strategies. Thus, the present study aimed to evaluate a conceptual model in a sample of Chinese adolescents in which cybervictimization would increase negative emotions, which, in turn, would increase NSSI. Then, the indirect association between cybervictimization and NSSI would be moderated by friendship quality (i.e., a moderated-mediation model). The friendship is a kind of social support, which provides support and company to friends, and the friendship quality is the measurement index that reflects the degree of friendship.

### The Mediating Role of Negative Emotion

General Strain Theory (GST) proposes that when individuals encounter environmental stimulation or stressful events (strain stimulation) and cannot solve it through existing experience or ability, they will experience a subjective feeling of being physiologically and psychologically oppressed. To alleviate this sense of oppression, they may use maladaptive behaviors (Agnew, 1992). From this theory, negative emotions are not only a result of strain stimulation but also a contributing factor for risky behaviors, which serves as a mediating variable between environment stimulation and behavior consequence. Existing research has shown that being bullied is a significant stressor, and adolescents who experience bullying may engage in NSSI (Claes et al., 2015). So, we propose that negative emotion might act as a mediator in the relationship between cybervictimization and NSSI among adolescents.

First, cybervictimization is often closely related to the victim’s negative emotions. Li (2015) compared cyberbullying to traditional bullying using a meta-analysis and found that the negative effects of cyberbullying on adolescents’ mental health were comparable to the effects of traditional bullying. Franks (2015) interviewed 10 adolescents aged 13–18 years and found that the emotional sequelae of cybervictimization included the loss of self-esteem and self-confidence as well as increased anger, embarrassment, and grief. When compared to adolescents with no experience of cyberbullying, adolescents who have experienced cyberbullying have higher levels of hostility, depressive symptoms, and anxiety related to persecution (Fan et al., 2018).

Second, managing negative emotions has been identified as a primary motivational factor for adolescents’ NSSI. The Experiential Avoidance Model (EAM) posits that the main function of NSSI is the avoidance of internal experiences or feelings that an individual does not want to experience (Chapman et al., 2006). Klonsky (2007) pointed out that the majority of research has viewed NSSI as a negative emotional coping style. Hughes et al. (2019) reported that among the numerous factors, negative emotions, such as anxiety and feelings of being overwhelmed, are the strongest predictors of adolescents’ NSSI. Behavioral experiments have also demonstrated that both guilt and shame could induce adolescents’ NSSI, while NSSI reportedly alleviated feelings of guilt and shame (Wang et al., 2019).

### The Moderating Role of Friendship Quality

Although the adolescents who experience frequent cyberbullying and have increased negative emotions might be at increased risk for NSSI, a positive correlation between the constructs has not been consistently shown (Kessel Schneider et al., 2012). This heterogeneity in outcomes might be contingent upon individuals’ social support that moderates the impact of cybervictimization on NSSI. The buffering model of social support proposes that social support, which is an important protective factor, could effectively reduce the adverse effects of stressful life events on adolescents’ mental health (Uchino, 2006). Several studies have indicated that social support buffers the relationship between adverse event life such as bullying and NSSI (Christoffersen et al., 2015; Claes et al., 2015; Alexandra et al., 2017). Shaw et al. (2019) found that social support plays a significant role in coping with bully effectively. Friendship quality is the measurement index of friendship, which reflects the support provided by friends, the degree of company, and the level of conflict (Parker and Asher, 1993). High friendship quality can not only provide external social support but also help to enhance their internal self-worth, so that adolescents can actively deal with setbacks and stress (Troop-Gordon et al., 2015). Mona (2017) found that peer social support was a potential protective factor for NSSI among adolescents.

When adolescents have emotional disorders due to life events, they might generate negative emotions such as loneliness, depression, and anxiety; however, high-quality friendship becomes a buffer for adolescents to resist negative emotions (Mounts, 2002; Kingery et al., 2011). This buffer then helps reduce the occurrence of externalizing behavior (Burk and Laursen, 2005; Tian et al., 2018). You et al. (2016) examined how peer group impulsivity moderated the individual-level relationship between depression and NSSI and found that friendship group pre-meditation weakened the relationship between individual depression and NSSI, while friendship group negative urgency strengthens the relationship between depression and NSSI.

### The Present Study

**Figure 1:**
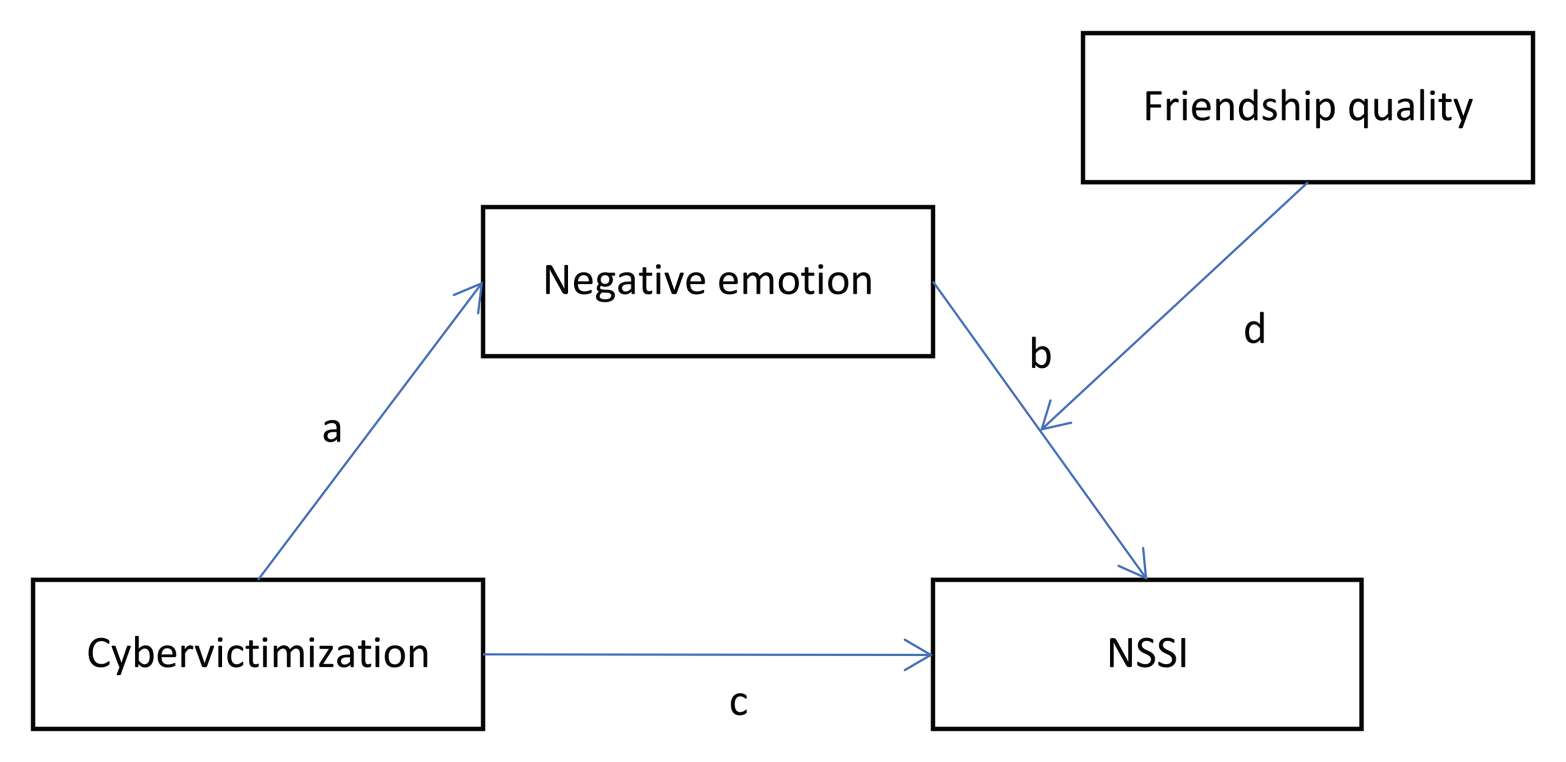
The proposed moderated-mediation model of the relationship between cybervictimization and non-suicidal self-injury (NSSI).

The purpose of the present study was twofold. First, this study examined whether negative emotion mediates the association between cybervictimization and NSSI in a sample of Chinese adolescents. Given existing research, we proposed the following for Hypothesis 1: Cybervictimization would positively impact adolescents’ negative emotions, which, in turn, would increase adolescents’ NSSI. In other words, negative emotions would mediate the association between cybervictimization and adolescents’ NSSI. Second, the study explored whether the indirect relationship between cybervictimization and NSSI *via* negative emotions was moderated by friendship quality. Based on the buffering hypothesis of social support and empirical evidence, we propose the following for Hypothesis 2: The indirect association between cybervictimization and NSSI *via* negative emotions would vary depending on the level of the adolescents’ friendship quality. Specifically, a high level of friendship quality would attenuate the path between negative emotion and NSSI. This study would contribute to an enhanced understanding the detrimental influences of cyberbullying on adolescents and provide insight regarding the development prevention strategies and methods of intervention. Figure 1 illustrates the proposed moderated-mediation model.

## Materials and Methods

### Participants

Participants were selected from two middle schools and two high schools in the Hunan province of China. Overall, 1,800 questionnaires were distributed. After excluding the invalid questionnaires, such as a pattern of irregular responses and blank questionnaires, 1,668 valid questionnaires were collected. Of these, 1,324 (79.38%) participants reported that they had been bullied on the Internet at least once in the last year. These participants were the focus of the present study. Among the adolescents experiencing cybervictimization, 727 (54.91%) were boys, 591 (44.65%) were girls, 6 (0.44%) were missing. The mean age of the participants was 13.67 years (*SD* = 1.34, range 11–17).

### Measures

#### Cybervictimization

Cyberbullying Scale was developed by Kwan and Skoric (2013) and translated and revised into the Chinese version by Chen et al. (2016), with items on both cyberbullying and cybervictimization, each of which included 17 questions, such as, “I have received threatening messages on social network sites.” The current study utilized the section that included the cybervictimization items. A 6-point rating scale was used in this questionnaire to rate items (1 = *never*, 2 = *once*, 3 = *twice to four times*, 4 = *five times to seven times*, 5 = *eight times to 10 times*, 6 = *more than 10 times*). The higher the total score, the more encounters with cyberbullying. In this study, the Cronbach’s *α* was 0.90.

#### Negative Emotions

The Negative Emotion Scale was compiled by Bradburn (1969) and revised by Chen and Zhang (2004) to measure children’s and adolescents’ negative emotions, including loneliness, depression, and irritability. The questionnaire consists of six items, such as, “I feel inexplicably annoyed.” Each item was rated on a 4-point scale, ranging from 1 (*no*) to 4 (*often*), with the total score serving as an indicator of negative emotions. Higher scores meant greater negative emotions. The validity of the scale has been demonstrated to be good. In this study, Cronbach’s *α* was 0.81.

#### Friendship Quality

Friendship Quality was measured using a scale initially developed by Parker and Asher (1993), which was then revised to create a brief version by Zou et al. (1998). The scale consists of 18 items, such as “We often help each other,” which represent six dimensions: affirmation and caring, intimate and exchange, companionship and recreation, help and guidance, conflict and betrayal, and conflict resolution. The items are rated on a 5-point scale ranging from *no coincidence* to *complete coincidence*, with the conflict and betrayal dimension using reverse scoring. The total score of friendship quality is obtained summing the ratings for each item, with higher scores indicating higher friendship quality. In this study, the Cronbach’s *α* was 0.85.

#### Non-suicidal Self-Injury

The Adolescent Self-Harm Scale was revised by Yu (2013) based on an existing scale (Feng, 2008) that assessed adolescents’ NSSI in contexts that do not involve suicidal intention. The questionnaire consists of 19 items, 18 of which measure two parallel components of NSSI – the frequency of NSSI and degree of physical harming behavior, such as “cut your skin intentionally,” and the last item was an open-ended question. The frequency items were rated on four levels: *never*, *once*, *twice to four times*, and *five times or more*, which were scored 0–3, respectively. The degree of physical harming items was rated on five levels: *none*, *mild*, *moderate*, *severe*, and *extremely severe*. The total score was the sum of the scores from the two parts, with higher scores indicating more severe NSSI. For this study, the Cronbach’s α was 0.93.

#### Covariates

Given that prior research has suggested that NSSI is correlated with the gender and age of adolescents (Barrocas et al., 2014; Li et al., 2019), we included these variables as covariates in the statistical analyses. Adolescent gender was dummy coded such that 0 = *male* and 1 = *female*.

### Procedures

This present study was reviewed and approved by the Institutional Review Board at the Institute of Psychology, Hunan Normal University. Data were collected in middle school classrooms between October and November in 2017. Undergraduate and graduate students in the psychology department were trained to collect the data and administered the questionnaires using standardized scripts that followed a procedure manual to ensure the standardization of the data collection process. Informed consent was obtained from the school administrators, adolescents, and their parents before data collection. The questionnaire was distributed in class. It took about 15 min to complete all the questionnaires. After all the participants answered, the questionnaire was collected. Every participant who completed all questionnaires received a ballpoint pen as incentives.

### Statistical Analyses

We used SPSS 19.0 to complete all statistical analysis. First, we calculated the descriptive statistics and bivariate correlations for our variables of interest and the control variables. Second, we used Hayes (2013) PROCESS macro (Model 4) to evaluate the mediating effect of negative emotions. Finally, we analyzed the moderated-mediation model using Hayes (2013) PROCESS macro (Model 14). All the continuous variables were standardized, and the interaction terms were computed from these standardized scores. The bootstrapping method produces 95% bias-corrected confidence intervals of these effects from 5,000 re-samples of the data. Confidence intervals that did not contain zero indicated significant effects. In all analyses, we included participants’ gender and age as covariates.

## Results

### Preliminary Analyses

Descriptive statistics and Pearson correlation coefficients for all study variables are presented in Table 1. The correlation matrix showed that cybervictimization in the sample of adolescents in China was significantly related to gender and age. Cybervictimization was negatively associated with gender (*r* = −0.12, *p* < 0.01) and positively associated with age (*r* = 0.08, *p* < 0.01). Moreover, cybervictimization was positively associated with NSSI (*r* = 0.30, *p* < 0.01), as was negative emotion (*r* = 0.22, *p* < 0.01). Finally, negative emotions were positively associated with NSSI (*r* = 0.22, *p* < 0.01).

**Table 1:** Means, SD, and correlation matrix (*N* = 1,326).

Variables	*M*	*SD*	1	2	3	4	5
1. Gender	0.45	0.50	-				
2. Age	13.67	1.34	0.04	-			
3. Cybervictimization	26.00	10.78	−0.12^**^	0.08^**^	-		
4. NSSI	3.40	11.81	−0.01	0.05	0.30^**^	-	
5. Negative emotion	14.10	4.14	0.18^**^	0.22^**^	0.22^**^	0.22^**^	-
6. Friendship quality	67.75	11.66	0.15^**^	−0.13^**^	−0.12^**^	−0.14^**^	−0.16^**^

Gender was dummy coded such that 0 = male and 1 = female.

* *p* < 0.05;

** *p* < 0.01.

### Testing for the Mediating Effect of Negative Emotions

The results are shown in Table 2. After controlling for gender and age, the bias-corrected bootstrap method indicated that the indirect effect of cybervictimization on NSSI through negative emotions was significant, ab = 0.04, *SE* = 0.01, 95% CI [0.02, 0.06], with the mediating effect accounting for 13.06% of the variance. Furthermore, the direct effect of cybervictimization on NSSI was statistically significant, c = 0.28, *SE* = 0.10, 95% CI [0.23, 0.034], *p* < 0.001. Therefore, the relationship between cybervictimization and NSSI was partially mediated by negative emotions.

**Table 2:** The mediating role of negative emotion.

Dependent variable (DV)	Mediating variable (MV)	Effect of cybervictimization on MV	Effect of MV on DV	Direct effect of cybervictimization	Indirect effect of cybervictimization	Total effect of cybervictimization
NSSI	Negative emotion	0.09	0.49	0.28	0.04	0.32
	95% CI	[0.06, 0.11]	[0.29, 0.66]	[0.08, 0.49]	[0.02, 0.06]	[0.11, 0.54]

NSSI, non-suicidal self-injury; DV, dependent variable; MV, mediating variable.

### Cybervictimization and NSSI: Testing for Moderated-Mediation

The results are shown in Table 3. It was determined that cybervictimization has a significant effect on negative emotion (*β* = 0.23, *SE* = 0.03, 95% CI [0.18, 0.28], *p* < 0.001), and the effect of negative emotions on NSSI was statistically significant (*β* = 0.16, *SE* = 0.03, 95% CI [0.11, 0.21], *p* < 0.001). Further, cybervictimization positively predicted NSSI (*β* = 0.24, *SE* = 0.03, 95% CI [0.18, 0.29], *p* < 0.001), and the interaction between negative emotions and friendship quality negatively predicted NSSI (*β* = −0.16, *SE* = 0.02, 95% CI [−0.21, −0.12], *p* < 0.001).

**Table 3:** The moderated-mediating effect of cybervictimization on NSSI.

	Negative emotions			NSSI		
	*β*	*t*	95% CI	*β*	*t*	95% CI
Gender	0.20^***^	7.75	[0.15, 0.25]	−0.01	−0.39	[−0.06, 0.04]
Age	0.19^***^	7.34	[0.14, 0.24]	−0.01	−0.53	[−0.07, 0.04]
Cybervictimization	0.23^***^	8.75	[0.18, 0.28]	0.24^***^	8.94	[0.18, 0.29]
Negative emotion				0.16^***^	5.95	[0.11, 0.21]
Friendship quality				−0.08^**^	−3.02	[−0.13, −0.03]
Negative emotion × friendship quality				−0.16^***^	−7.00	[−0.21, −0.12]
*R*^2^	0.12			0.39		
*F*	63.96^***^			40.22^***^		

* *p* < 0.05;

** *p* < 0.01;

*** *p* < 0.001.

The bias-corrected bootstrap method indicated that the indirect effect of cybervictimization on NSSI, through negative emotion, was moderated by friendship quality, with the index of moderated mediation being −0.04, 95% CI [−0.09, −0.002]. When the level of friendship quality was higher (i.e., 1 *SD* above the mean), the mediating effect of negative emotions in the relationship between cybervictimization and NSSI was not significant, with the index of the mediating effect being −0.001, 95% CI [−0.05, 0.03]. Meanwhile, when the level of friendship quality was lower (i.e., 1 *SD* below the mean), there was a mediating effect of negative emotions in the relationship between cybervictimization and NSSI, with the index of the mediating effect being 0.07, 95% CI [0.03, 0.13].

**Figure 2:**
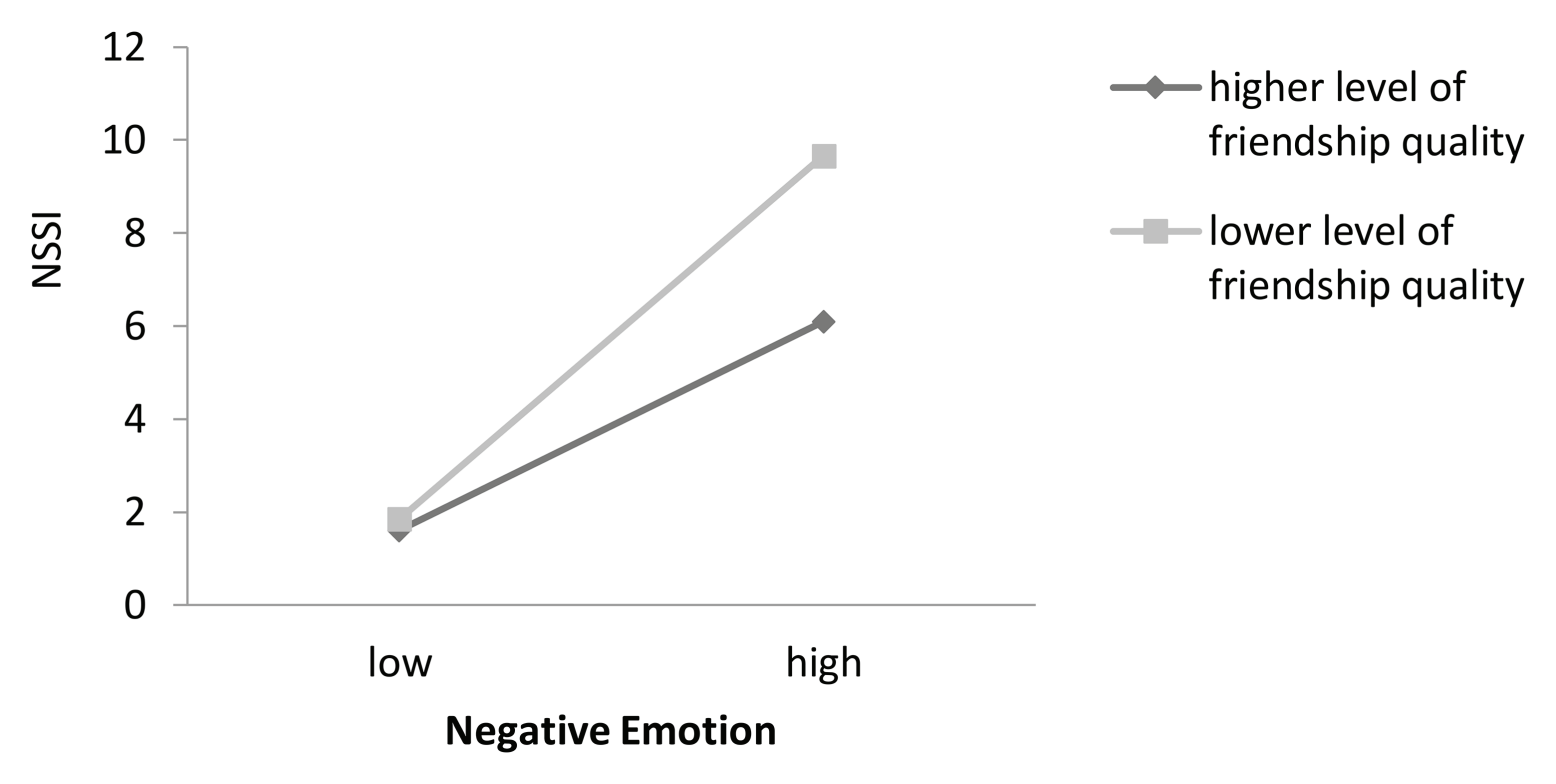
Model of the test for simple slopes showing the moderating influence of friendship quality of the association between negative emotions and NSSI.

Following the procedures suggested by Aiken and West (1991), the present study examined the predictive effect of negative emotion on NSSI by conducting separate examinations for the high (+1 *SD*) and lower level of friendship quality in order to illustrate the nature of the moderating effect further. The simple slope test indicated that when friendship quality was higher, higher levels of negative emotion were associated with more NSSI (*β*_simple_ = 0.27, *p* < 0.01). Furthermore, when friendship quality was lower, the effect of negative emotion on NSSI was much stronger (*β*_simple_ = 0.37, *p* < 0.001; Figure 2).

## Discussion

Cyberbullying is a form of bullying, which has been found to contribute to stress and mental pressure in those who are victimized by this behavior, and it can lead to numerous psychological and behavior problems, including NSSI (Hay and Meldrum, 2010; Kessel Schneider et al., 2012). NSSI is a maladaptive coping style (Sornberger et al., 2013) that is closely associated with various psychiatric experiences (Honings et al., 2016). To date, cyberbullying has been a relatively understudied public health problem in adolescents (Westers and Culyba, 2018). This study developed and evaluated a moderated-mediation model, to help clarify “how” and “for whom” cybervictimization are associated with NSSI.

### The Mediating Role of Negative Emotions

Hypothesis 1 stated that negative emotions would mediate the relationship between cybervictimization and NSSI. Our study found that cybervictimization was associated with increased negative emotions, which, in turn, was associated with NSSI in our sample of Chinese adolescents. In other words, negative emotions mediated the relationship between cybervictimization and NSSI, which supported Hypothesis 1. Thus, an increase in negative emotions may serve as one explanatory mechanism for the relationship between cybervictimization and adolescent engagement in NSSI. As far as we are aware, this study is the first to report such a model. Our research provides support for General Strain Theory (Agnew, 1992), which has proposed that stressors regarding environment stimulation that an individual experiences or their life events are unrelated to NSSI, aggression, and other risky behaviors contribute to the development of depression, anxiety, anger, and other negative emotions. NSSI, aggression, and other risky behaviors are coping mechanisms that are designed to alleviate negative emotions. Cybervictimization serves as a stressor that evokes victims’ negative emotions, leading victims to employ NSSI as a coping strategy.

Our study is also consistent with NSSI’s Experiential Avoidance Theory (Chapman et al., 2006), which suggests that the mechanism leading to the formation of NSSI is a situation that evokes the individual’s negative emotions that leads to individuals perform NSSI as a means of evading or relieving unhappiness emotional experiences. The consequence of NSSI, which is the relief of negative emotions, brings immediate gratification to individuals. This negative reinforcement then strengthens the association between the stimulation of unhappiness and NSSI. Thus, the subsequent experience of unpleasant emotional experiences by an individual leads to NSSI becoming an automatic escape response.

In addition to the mediation results, each of the individual pathways in our mediation model is noteworthy. First, cybervictimization was found to have a significant direct effect on both negative emotions and NSSI. This demonstrates the various negative effects of cybervictimization on adolescents and provides empirical support regarding the harmfulness of cyberbullying. Compared with traditional bully, cyberbullying is easier to reach, as well as allowing invisibility and anonymity (Tokunaga, 2010). If adolescents lack sufficient social support or good psychological adjustment ability, they are more likely to develop psychological and behavior problems. Second, negative emotions are a significant positive predictor for adolescents’ NSSI. The predictive effect of negative emotions on NSSI has been reported in numerous studies, which suggests that NSSI acts as a method for managing emotions (Klonsky, 2007); however, NSSI is unable to resolve all of adolescents’ emotional problems. Wang et al. (2019) found that, although NSSI can relieve the psychological confusion caused by negative emotions, in the long term, NSSI and negative emotions are mutually perpetuating. Thus, if NSSI alleviates the negative emotions caused by repeated cyberbullying over an extended period, the negative emotions and NSSI might begin to mutually reinforce each other.

### The Moderating Role of Friendship Quality

The present study also confirmed the moderating role of friendship quality in the indirect association between cybervictimization and adolescent NSSI. Specifically, we found that friendship quality attenuated the relationship between negative emotions and NSSI. The regulation of the mediating effect of friendship quality on negative emotions is achieved through regulating the relationship between negative emotion and NSSI. Compared to those adolescents with lower friendship quality, those with high friendship quality who experience being cyberbullied have the relationship between negative emotions and NSSI weakened. Thus, adolescents with high friendship quality are less likely to regulate the negative emotions caused by cybervictimization through NSSI. Therefore, Hypothesis 2 was supported.

Friendship reflects the attachment to a companion through an intimate emotional relationship. Whether in the real world or the virtual world, adolescents express a strong need of belonging (Fabris et al., 2020; Longobardi et al., 2020). For social functioning of adolescents, peer relationships are even greater important than family relationships (Badenes-Ribera et al., 2019). Research has demonstrated that adolescent attachment to a best friend has a stronger relationship to depression, self-esteem, self-ability, and attitudes toward learning better than attachment to a general companion or peer group (Wilkinson, 2010). Adolescents who have good relationships with friends have lower levels of depression, anxiety, loneliness, and other negative emotions (Woods et al., 2009; Kim, 2015), as well as fewer problem behaviors (Kawabata et al., 2010). Friendship appears to effectively alleviate negative emotions and problem behaviors (Sinanan and Gomes, 2020). The emotional management function of NSSI emphasizes its role in serving to manage negative emotions for the purpose of emotional control (Messer and Fremouw, 2008). When adolescents have high-quality friendships, they are more likely to reduce psychological distress by pouring out or seeking help instead of NSSI. In this sense, emotional support provided by high-quality friendships acts as a more adaptive form of emotional management that can replace nonadaptive coping styles, such as NSSI.

In summary, this study identified a significant moderated-mediation model that explained the effect of cybervictimization on adolescents’ NSSI, which integrated the General Strain Model and the Buffer Model. Our proposed model provides information on the relationship between cybervictimization and adolescents’ NSSI and offers corresponding empirical evidence for theories regarding the experiential avoidance of NSSI along with offering some potential methods for preventing the deleterious consequences of cyberbullying for adolescents. Further, this model addresses the critical issue of “what works for whom,” by revealing that negative emotions area primary mediating mechanisms and adolescent friendship quality is one variable that can account for the heterogeneity in the relationship between cybervictimization and adolescents’ NSSI.

### Limitations and Practical Implications

There are several limitations to this study. First, due to the study’s cross-sectional design, we cannot make any causal inferences about observed associations, nor can we reveal the role of developmental factors. Future studies should use longitudinal research to better pinpoint the paths in our theoretical model. Second, although self-report has been widely used in the literature to assess both cybervictimization and NSSI, this method of data collection has some inherent disadvantages, such as strong subjectivity, which could lead to some deviations on data inevitably. Future studies should include multiple methods of data collection (e.g., adolescent, parent, peer, or teacher report) to allow for mutual confirmation and the acquisition of more objective and accurate data. Finally, during adolescence, there are different manifestations of NSSI (Yu, 2013); therefore, future studies should investigate the mechanisms of cybervictimization on different forms of NSSI to obtain more accurate research results.

Despite these limitations, our findings have important practical implications. First, given the universality and harmfulness of cybervictimization for adolescents, we need to emphasize the need to understand the experiences of cybervictimization on adolescents. Intervention and prevention programs should be used as much as possible to reduce cyberbullying (Gaffney et al., 2019). Second, based on the General Strain Model, we verified the mediating effect of negative emotions on the relationship between cybervictimization and NSSI. This pathway suggests that by teaching emotional management skills to adolescents who are dealing with cybervictimization, they can learn to effectively deal with the emotional distress caused by the experience of cyberbullying and also prevent the development of problem behaviors. Third, our study found that high-quality friendship can attenuate the influence of negative emotions on NSSI. However, for adolescents, especially in China, tremendous academic pressure can lead to little time to devote to their friendships, and the emphasis on academic competition created challenges for the formation of high-quality friendships. Thus, schools can assist with managing the phenomenon of cyberbullying by promoting adolescent peer relationships and encouraging the cultivation of high-quality friendships. Schaefer et al. (2011) found that involvement in positive extracurricular activities can promote the formation of adolescent friendships and have a positive effect on their psychological health. Perhaps, schools can also consider increasing the diversity of school life beyond academics to further enhance adolescents’ ability to cope effectively with stressful life events.

Although further replication and extension are needed, this study serves as an important step in understanding how cybervictimization is related to adolescent NSSI. Using General Strain Theory and the buffering model of social support, our research constructed a moderated-mediation model and verified the mediating influence of negative emotions on the relationship between cybervictimization and NSSI, and the moderating effect of friendship quality on this mediating effect. This model has not been previously studied, and it helps extend the field’s understanding of the negative effects and mechanism of cybervictimization and identifies possible means of reducing the negative effects of cyberbullying on victims.

## Data Availability Statement

The raw data supporting the conclusions of this article will be made available by the authors, without undue reservation.

## Ethics Statement

The studies involving human participants were reviewed and approved by IRB, Institute of Psychology, Hunan Normal University. Written informed consent to participate in this study was provided by the participants’ legal guardian/next of kin.

## Author Contributions

YW: substantial contributions to the design of the work, data analysis, and writing. AC: substantial contributions to the design of data analysis and writing. HN: substantial contributions to the design of the work and language polish. All authors contributed to the article and approved the submitted version.

### Conflict of Interest

The authors declare that the research was conducted in the absence of any commercial or financial relationships that could be construed as a potential conflict of interest.
